# Exploring item comprehension and evaluation in the SELC scale: a qualitative pre-testing study

**DOI:** 10.1080/02813432.2026.2663295

**Published:** 2026-04-27

**Authors:** Marie Rask, Sara Alenius, Lina Behm, Marie Nilsson, Petra Nilsson Lindström

**Affiliations:** ^a^ The Research Groups: PRO-CARE, FOHRK and FPHIS, Department of Nursing and Integrated Health Sciences, Faculty of Health Sciences, Kristianstad University, Kristianstad, Sweden; ^b^ The Research Group MHS, Department of Nursing and Integrated Health Sciences, Faculty of Health Sciences, Kristianstad University, Kristianstad, Sweden

**Keywords:** Content validity, counselling, lifestyle, scale development, SELC 20 + 20, qualitative pre-testing, questionnaire evaluation

## Abstract

**Background:**

Lifestyle counselling is part of healthcare professionals’ work, but when focused on individual lifestyle counselling, its effects are often limited or context-dependent, highlighting the need for a person-centered approach where motivation, readiness for change, and relational aspects are key. The SELC scale measures self-efficacy, relevant to care professionals’ engagement, but interpretation needs further study. The aim of this study was to explore how participants understood and evaluated items in the SELC scale and to identify potential issues related to clarity, relevance, and response options through qualitative pre-testing interviews.

**Methods:**

Qualitative pre-testing interviews with eight strategically selected participants explored how SELC items were understood and evaluated, using a structured guide with probing, and were analyzed with deductive qualitative content analysis.

**Results:**

Participants found most items clear and easy to understand, although some terms were perceived as ambiguous. Items were generally perceived as relevant and consistently structured. Response alternatives were regarded as easy to distinguish, and the instructions were clear and straightforward. However, the open-ended item was unclear, and comments suggested that item order and the expert-driven perspective reduced the instrument’s practical usefulness. In the revised SELC 20 + 20, terminology was clarified, items neutrally phrased, sequence aligned with real-life conversations, and visual and structural consistency improved.

**Conclusions:**

Pre-testing interviews identified strengths and improvement areas in the SELC scale and guided its refinement. Combined with previously published psychometric testing, results suggest the SELC scale has potential for assessing self-efficacy among healthcare professionals and in educational settings, although further validation is needed.

## Background

Lifestyle counselling, including advice and support regarding physical activity, eating habits, tobacco use, and alcohol consumption, plays a crucial role in the prevention and management of non-communicable diseases (NCDs), such as cardiovascular disease, type 2 diabetes, and certain cancers [1]. As these conditions are among the leading causes of morbidity and mortality globally, addressing modifiable lifestyle behaviours is a central public health priority. In Sweden, the National Board of Health and Welfare [2] mandates that healthcare professionals provide evidence-based lifestyle counselling, based on national guidelines, as an integral part of healthcare services aimed at preventing and managing unhealthy lifestyle behaviours.

Although unhealthy lifestyle habits are associated with an increased risk of non-communicable diseases, there is strong evidence that such behaviours are strongly related to socio-economic conditions, such as income, educational level, and living circumstances. Structural and policy-level interventions have been shown to be most effective in prevention work, whereas individual-level lifestyle interventions tend to have limited or context-dependent effects. Individual lifestyle counselling within healthcare should therefore primarily be understood within a person-centered context of prevention work, where motivation, readiness for change, and relational aspects of care are central [3].

Despite the recognised importance of preventive work and the existence of clear guidelines, implementation in clinical practice remains inconsistent. For instance, a Swedish study involving over 52,000 patients revealed that only 30% had received any form of lifestyle counselling from healthcare professionals, although 40% of those who did receive advice reported making lifestyle changes [4]. This gap between recommendations and practice reflects broader challenges reported in the literature. Healthcare professionals cite a range of barriers to providing lifestyle counselling, including time constraints, limited training, perceived ineffectiveness of interventions, and concerns about patient receptiveness [5–7]. Physicians report obstacles such as inadequate training [8,9], lack of time [5], doubts about the effectiveness of their interventions, and low personal interest in lifestyle issues [10]. Nurses also cite insufficient counselling skills and a lack of confidence as significant challenges [11,12]. In one Finnish study, only about half of the participating doctors and nurses felt they had adequate knowledge to provide effective counselling [13]. In addition, healthcare professionals’ own lifestyle habits, interest in lifestyle issues, and lack of confidence have been shown to influence whether they engage in lifestyle counselling [9,10,14].

One of the most significant psychological factors influencing the willingness and ability of healthcare professionals to engage in lifestyle counselling is self-efficacy, i.e. the belief in one’s ability to perform specific tasks successfully [15,16]. Self-efficacy theory, as defined by Bandura [15], emphasises that individuals’ confidence in their capabilities directly influences their motivation, perseverance, and decision to engage in particular behaviours. According to Bandura, self-efficacy is shaped by four primary sources: mastery experiences (direct performance of a task), vicarious experiences (observing others succeed), verbal persuasion (encouragement from others), and emotional and physiological states. Within healthcare, higher self-efficacy has been linked to more frequent and effective implementation of lifestyle counselling [16]. Conversely, low self-efficacy may result in avoidance of counselling tasks, stemming from self-doubt or concerns about patient resistance.

To enhance healthcare professionals’ readiness for counselling, interventions that promote self-efficacy, through practical experience, observation, feedback, and emotional support, have shown promising results [17,18]. Educational programmes that incorporate these elements can significantly improve both self-efficacy and competence [18]. In parallel with such educational initiatives, valid and reliable instruments are needed to measure healthcare professionals’ readiness and self-efficacy in lifestyle counselling. The successful implementation and sustained application of lifestyle counselling in healthcare settings depend not only on training and policy support but also on ongoing evaluation [19]. Continuous assessment is essential for ensuring the quality of counselling practices, identifying barriers in real time, and supporting ongoing improvement [20]. Regular evaluation enables healthcare professionals and organisations to refine their approaches, adapt to evolving patient needs, and maintain adherence to evidence-based guidelines [21].

To address the lack of validated instruments for assessing self-efficacy in lifestyle counselling, we developed the Self-Efficacy in Lifestyle Counselling (SELC) scale. The SELC scale evaluates healthcare professionals’ self-efficacy across four key lifestyle domains: physical activity, eating habits, tobacco use, and alcohol consumption. Its development followed a rigorous process, including item generation based on national guidelines, expert review, and psychometric testing, resulting in demonstrated reliability and validity. While the tool shows strong psychometric properties [22], further investigation is needed to explore how participants understand and evaluate its items. Examining item clarity, relevance, and suitability from the perspective of different target groups is essential to ensure that the instrument adequately represents the intended construct and supports evidence of content validity [23].

A central aspect of instrument development concerns ensuring that items adequately represent the intended construct and are understood as intended by the target group. Within a functional approach to content validity, the degree to which items are perceived as clear, relevant, and representative of the construct is considered essential for supporting valid interpretations of responses [23]. Thus, examining how potential participants understand and appraise questionnaire items constitutes an important component of the validation process.

Qualitative pre-testing is widely recommended in early stages of instrument development to identify ambiguities, misunderstandings, and weaknesses in wording, response formats, or content coverage [24]. By systematically exploring how items are understood and evaluated by individuals with relevant experience, researchers can refine instruments and strengthen evidence related to content validity.

Gaining insight into these processes can inform the refinement of instruments such as the SELC scale and enhance the applicability in both research and clinical practice. The aim of this study was therefore to explore how participants understood and evaluated items in the SELC scale and to identify potential issues related to clarity, relevance, and response alternatives through qualitative pre-testing interviews.

## Methods

### Study design

This study forms part of the quality testing of the SELC scale, which measures healthcare professionals’ confidence in their ability to counsel patients or clients on lifestyle habits. The present study reports qualitative results from qualitative pre-testing interviews [24] conducted as part of the SELC scale’s quality testing. These interviews aimed to strengthen the instrument’s future usability in practice by exploring how its items are interpreted and evaluated by individuals with substantial experience and expertise in the field, with particular attention to clarity, relevance, and overall suitability of the items. By systematically analyzing participants’ reflections, the study contributes evidence related to the instrument’s content validity, understood as the degree to which items adequately represent the intended construct [23]. Qualitative pre-testing constitutes a recommended step in instrument development to refine wording, identify ambiguities, and improve item relevance as a complement to psychometric testing [24].

### Instrument

This study formed part of the development of the SELC 20 + 20 instrument [22]. The qualitative pre-testing interviews were an integral part of the process to condense and improve both the individual items and the overall structure of the instrument. The development process included two phases. In the first phase, ten interviews were conducted and analysed to identify areas in need of revision. Based on these results, the instrument was modified. The data for the present study were collected during the second phase, in which an additional eight interviews were conducted using the revised version, referred to as SELC 28. At this stage, the instrument was organised into two constructs: knowledge and ability, each consisting of 20 items. Within each construct (knowledge and ability), the 20 items were divided into four domains of lifestyle habits: tobacco use, alcohol consumption, physical activity, and eating habits. The instrument also includes six items related to learning methods, a separate domain that is not included in the present study. Additionally, it contains two open- ended questions: one placed after the last item on dietary habits and another after the last item on learning methods. All other items, assessing the participant’s self-efficacy in lifestyle counselling, were answered using a 4-point Likert scale. The response alternatives ranged from ‘very insecure’ to ‘very sure’ regarding tobacco use, alcohol consumption, physical activity, and eating habits, and from ‘not at all’ to ‘a very high degree’ regarding learning methods. For a comprehensive overview of SELC 28 (see Additional File 1).

### Setting and participants

A qualitative pre-testing interview study [23,24] was conducted between November 2022 and January 2023 with university nursing students, university teachers in health-related subjects, and healthcare clinicians in southern Sweden. These groups were selected because this study aimed to explore how participants understood and evaluated items in the SELC scale and to identify potential response-related issues. Sample size in pre-testing studies may vary according to context but typically ranges from 5 to 15 participants [24]. In this study, eight participants were interviewed. Participants were selected strategically based on relevant characteristics, for example, gender and profession [25]. Therefore, the sampling was purposeful, and the criterion for inclusion was either being a university nurse student, university health-related teacher, or healthcare clinician. Participants were considered individuals with relevant experiential knowledge who could contribute to assessing the instrument’s clarity and relevance. The criterion aimed to ensure a wide range of participants (see Table 1). The university students were recruited from a nursing programme (year 1, semester 2). The clinicians were recruited from a knowledge centre for lifestyle counselling. One of the authors (SA) recruited both students and clinicians. The university teachers were recruited from a nursing programme (bachelor) and from a public health programme (bachelor) by all the authors.

**Table 1.** Characteristics of the participants.

Characteristics	*n* = 8
Age	
Median years (range)	41.5 (32)
Gender	
Men	3
Women	5
Profession	
Nurse student	2
University health-related teacher	2
Healthcare clinician	4
Education	
Dietitian	1
Public health practitioner	1
Primary care nurse	2
Physician	1
Registered nurse	1
Nurse student	2

Those who agreed to participate received an informational letter about the study and its ethical considerations, either sent by e-mail or in paper form. The same information was also repeated orally before the interview began. Written or oral informed consent was obtained from all participants before the interview started. The study was approved by the Ethical Board at the Faculty of Health Sciences, Kristianstad University in Sweden, Dnr: U2022-2.1.12-2112.

### Procedure

The qualitative pre-testing interviews were conducted by three of the authors (MR, SA, and LB). Interviews were held either in person or digitally via Zoom and lasted between 27 and 81 min, with a mean duration of 54 min. All interviews were recorded and transcribed verbatim afterwards. The interview guide included background questions on age, gender, profession, and educational background, followed by eight target questions (see Table 2) and associated probing questions. At the beginning of the interviews, participants were informed that the purpose of the interviews was to explore how they understood, reflected on, and evaluated the questionnaire items, and to identify any items that might be unclear, ambiguous, or in need of revision [24]. They were also informed that follow-up questions would be asked to encourage them to elaborate on their thoughts, interpretations, and reflections when responding to the items. The interview was conducted in a stepwise manner: the interviewer presented a question, the participant provided an answer, and the interviewer then asked clarifying or elaborating questions based on the response. This process was sometimes repeated to obtain a more detailed understanding of the participant’s perspective. Examples of follow-up questions included how specific words in each item were understood, participants’ positive and negative reflections when answering, and whether the items were perceived as relevant to their professional or educational context. The focus was to identify potential comprehension issues and gather evaluative feedback on item quality and relevance. As the SELC 28 includes four lifestyle-related domains (excluding the learning methods domain), the same target questions were asked for each domain. The interviews began by asking participants to describe any previous experience in providing lifestyle counselling; their general attitude towards surveys, and the reasons why they chose to answer, or not answer, a survey when invited to a survey. The interview then explored participants’ understanding and interpretation of the items, their evaluations of item clarity and relevance, and how they perceived the response alternatives. The participants did not need to tell the interviewer their final selected response option, as individual answers were not the focus of this study.

**Table 2.** The interview guide for the individual interviews.

**Questions about the instrument**
**Questions relating to each lifestyle habit**
To what extent do you find the items relevant?
Were any items unclear or difficult to interpret?
Do you consider any important items to be missing?
To what extent are the response options appropriate and relevant?
Are the response options sufficiently clear and easy to distinguish?
**Questions about the instrument in general**
What is your overall impression of the instrument?
How would you assess the layout and visual design of the instrument?
How clear and helpful are the instructions provided in the instrument?

### Analysis

To systematically analyse the interview data, a deductive qualitative content analysis was conducted, in accordance with Elo and Kyngäs [26]. A manifest content analysis was used, focusing on the explicit content of participants’ reflections and evaluations of the instrument. The analysis was performed using a developed categorization matrix, structured around two overarching categories: *Item comprehension and interpretation* and *Participants’ evaluations of item quality and relevance*. The category *Item comprehension and interpretation* captured participants’ descriptions of how they understood the wording of items, interpreted key concepts, and reflected on the meaning of the questions. This category encompassed two subcategories: understanding and interpreting items and key concepts, and the influence of prior experiences and knowledge. The category called *Participants’ evaluations of item quality and relevance* contained participants’ appraisals of item clarity, relevance, response alternatives, and layout. This category encompassed three subcategories: appraisal of clarity and relevance, appraisal of response alternatives, and appraisal of layout. Furthermore, before conducting the content analysis, a quality control procedure was carried out by the first author (MR). This process entailed a thorough review of the transcribed interviews. During this process, an ‘X’ was marked in a cross-tabulation table for each interview in separate documents. The purpose of this process was to obtain an overall picture of the data and to synthesise a comprehensive overview of the results from each interview, separately. The cross-tabulation then served as a basis for discussions with the other researchers.

As part of the content analysis, the first author (MR) initially re-read all interview transcripts and coded the data according to the categorization matrix. The coding was carried out in separate documents, each corresponding to one of the categories. The other researchers were then each assigned these documents to review. The purpose was to review the data to identify any excerpts that may have been misclassified. This was followed by a meeting to discuss potential reassignments of data. Finally, consensus was achieved regarding the categorization of all data under the two categories. This analytical approach enabled a structured examination of both how the items were understood and how they were evaluated, thereby informing the ongoing refinement of the instrument.

## Results

The results are structured according to two overarching analytical categories labelled *Item comprehension and interpretation* and *Participants’ evaluations of item quality and relevance.* Both categories were represented across all interviews. These categories were used to organize participants’ reflections and to identify issues related to how items were understood and interpreted, as well as how they were appraised in terms of clarity, relevance, response alternatives, and layout. Together, the results illuminate aspects central to qualitative pre-testing in instrument development and contribute to evidence of content validity [23,24]. Table 3 presents exemplary quotations from participants, referenced in the text by numerical identifiers (quotation ID; QID).

**Table 3.** Subcategories and quotations by participants and quotation IDs.

Subcategory	QID	Quotation
Understanding and interpreting items and key concepts	1	[…] mapping different lifestyle habits but not everything. It’s not always that I find a risky use. […] no, so in a way I think. Enough that it becomes […] well, wrong to use mapping. (R:13)
	2	[…] mapping might instead identify […] if it is about the professional being able to identify possible tobacco use in an individual, then identify is a better word. Then we are kind of OK at the individual level […]. (R:14)
	3	[…] there is a difference between knowledge and ability […] I’m a little afraid that they will just be equated. (R:11)
	4	[…] confidence in knowledge means that you have scientifically based and theoretical knowledge about the subject […] I can kind of talk about it and feel that I know what I’m talking about […]. Ability, then I interpret it as the ability to have a conversation about the purely practical […]. (R:15)
	5	[…] if it is some kind of illness, symptom or diagnosis. So, it is that connection, I think, that often influences the dialogue, so it becomes a little more than just giving advice. (R:17)
The influence of previous experiences and knowledge	6	[…] I know what I can do and what I am capable of after more than 22 years in primary care. A lot of what I base this on is my experience in talking about lifestyle habits. Yes, I have training in that too, both regarding tobacco cessation and MI. (R:18)
	7	[…] it’s easy to answer the questions, and this is largely because of the fact that I have worked on privately; it’s not something I learned through any school but my own life experience. It’s because I myself have had a risky use of alcohol and an unhealthy use of tobacco and very low physical activity. […]. (R:16)
	8	[…] then you start talking about what nicotine does to all the substances in tobacco and how they can affect the body. Then it’s more factual. […] then, MI strategies are used to provide motivation. (R:18)
	9	[…] I can make an assessment, but if it is correct, that is why I rate myself lower. (R:16)
Appraisal of clarity and relevance	10	[…] yes, absolutely the questions are relevant. Yes, to lifestyle. (R:12)
	11	[…] would then be the health benefits of healthy eating habits, reduces the health risks associated with unhealthy eating habits. It may be good to distinguish between health promotion and disease prevention. (R:11)
	12	[…] because I might feel a bit uncertain about what to write. I always think it’s good to have some free text space, because if you’re kind of committed, it’s good to know that you can add comments. (R:17)
Appraisal of response alternatives	13	I think it’s good that we must take a stand. […] so that we’re being pressured into it. That there’s no five, so you choose three. (R:12)
Appraisal of layout	14	I thought I managed to remember the ones up there, what each one is and what they represented, sort of. But it becomes clearer if the extremes are written down on each line. I generally like it, because you don’t have to go up and check what four meant, what one meant, but it all follows along. (R:14)
	15	[…] for me, it would have been logical if it had followed the order in which the conversation takes place. I start with a survey, then assess the person’s motivation, then discuss the health benefits, then I give advice and this about motivational strategies is what happens last. It’s the kind of thing that my brain can think of. (R:13)
	16	[…] the design is nice. I’m very picky about that. I studied design and aesthetics, yes, but it’s great. […] I think the design and layout are very clear. There’s nothing you should change. (R:12)

QID: quotation ID.

Several changes and improvements were made based on participants’ reflections, considering their feasibility and applicability. For an overview of the identified strengths and weaknesses, as well as the implemented improvements (see Table 4). As a result of these revisions, the instrument was finalised and named SELC 20 + 20.

**Table 4.** Overview of strengths, weaknesses, and improvements organized by the overarching categories: item comprehension and interpretation and participants’ evaluations of item quality and relevance.

Categories	Strengths	Weaknesses in SELC28	Improvements in SELC20 + 20
Item comprehension and interpretation		Some terms are complex	‘Mapping’ changed to ‘Identifying’
		Hard to distinguish between knowledge and ability	The words ‘theoretical’ (knowledge) and ‘practical’ (ability) were added
		Uncertainty might lead to over- or underestimation of capabilities	
Participants’ evaluations of item quality and relevance	Items are relevant	Additional lifestyle habits could be added	
	Similarity of items on each lifestyle habit	Awkward but functioning structure of some items	The wording of some items was changed
	Even numbers of response alternatives	Some items are framed positively/negatively	Items posed neutrally
		Expert-driven perspective	
		The open-ended items are ambiguous	The first open-ended item was removed and the second one rephrased
	Response alternatives are relevant and easy to distinguish	Order of items should reflect real-life conversations	The order of item 3 and 4 was changed in all parts (tobacco/nicotine use, alcohol consumption, physical activity and eating habits)
	Concise and clear instructions		
		More items would add value	
		Design inconsistencies	Consistent design throughout the instrument

### Item comprehension and interpretation

This category captured results related to the participants’ cognitive processes, such as understanding of the items and whether there is congruence between the intended meaning and their interpretation.

#### Understanding and interpreting items and key concepts

The results indicate that participants generally felt they understood the intended meaning of the items and their key concepts. Participants often incorporate a third party into their reflections, namely, the individual they would counsel on lifestyle habits. In interpreting the items, they draw on past experiences or hypothetical scenarios in which they provided such guidance. Although participants felt they understood the intended meaning of the items, they acknowledged the complexity of certain concepts and expressed doubts about whether their interpretations aligned with what was intended. The results indicate that participants critically examined their understanding of various terms, discussing possible interpretations and alternative terminology. They also recognised the challenge of selecting terms that are both clear and resistant to multiple interpretations.

One term identified as complex was ‘mapping’ (QID1). One interpretation suggested that mapping involves a structured framework, where predefined items are systematically checked off. This term was viewed as reflecting a more large-scale, positivistic approach, focusing on groups rather than the individual. An alternative term, ‘identifying’, was proposed, emphasizing a relational aspect where lifestyle habits are explored collaboratively through dialogue (QID2). Through such conversations, potential risk behaviours or unhealthy habits (e.g. insufficient physical activity or poor dietary choices) can be recognised. The term ‘identifying’ thus conveys a more joint, exploratory process rather than an objective mapping.

A key concept in the questionnaire, ‘knowledge’, was also highlighted as complex, particularly in relation to ‘ability’ (QID3). At the start of the questionnaire, an explanatory description is provided outlining how ‘confidence in knowledge’ and ‘confidence in ability’ should be understood, which participants found helpful for interpreting subsequent items. Despite this clarification, participants reported difficulty in clearly distinguishing between the two terms. Knowledge was perceived as having a foundational understanding of the subject, i.e. being well-informed and feeling confident when discussing it. Ability, in contrast, was seen as the capacity to apply this knowledge in practice. This distinction was perceived as potentially challenging, particularly for participants lacking experience or who had not performed a specific task for a long time. This uncertainty was exacerbated by ambiguities in the wording of certain items, especially regarding whether knowledge referred to personal knowledge or a broader, general understanding. Participants suggested using words such as ‘my’ or ‘your’ to make it clearer that the items referred to individual abilities and knowledge. Without such clarifications, there is a risk of conflating the two terms, leading to confusion and uncertainty about how to respond. Such modifications could make the questionnaire more user-friendly and help participants feel more confident in their answers.

Participants highlighted another challenge in distinguishing between knowledge and ability. When knowledge is understood as theoretical, scientifically grounded, and acquired through formal study, it is more clearly distinguishable from ability. However, when knowledge is conceptualised as including more than theoretical understanding, such as tacit knowledge, distinguishing it from ability becomes more challenging. Applying practical skills, such as conducting counselling sessions, simultaneously develops both knowledge of the specific subject and the practice of counselling itself. Within this broader understanding, knowledge and ability become interdependent and mutually influential (QID4).

The terms ‘advice’ and ‘motivational strategies’ were also discussed in relation to applying knowledge. One possible interpretation of giving advice was as a one-way communication, where health professionals provide facts and information about lifestyle habits. However, participants suggested an alternative understanding of advice as a relational process. In this view, advice is not merely the transmission of general facts but is instead tailored to the specific health benefits relevant to the individual. For instance, instead of advising someone simply to quit smoking, advice might focus on how quitting could improve a wound that is not healing (QID5). This understanding of advice was seen to overlap with the concept of motivational strategies.

The results underscore the importance of situating the questions within context, where the person being counselled becomes a key consideration. A term or questionnaire item may be understood and interpreted differently depending on the situation. For example, the understanding of ‘alcohol risk consumption’ may vary depending on the individual being counselled. If the individual is a teenager or a pregnant woman, any alcohol consumption would be considered risky. In contrast, a different assessment might apply to an adult experiencing anxiety or distress, while yet another perspective may be relevant for a seemingly healthy adult. Although an established definition of risk consumption exists, health professionals must adapt their interpretation to the specific situation and the individual with whom they are engaging. This situational sensitivity introduces complexity into the understanding of risk consumption and requires both flexibility and contextual awareness from the professional.

#### The influence of previous experiences and knowledge

In the process of understanding and interpreting the items, the participants reflected on past experiences and retrieved previous knowledge. This was used to capture issues related to the basis on which participants formed their responses, rather than to map actual memory processes. Their reflections suggest the use of two main approaches, applied either consciously or unconsciously. The first is an approach whereby participants draw upon relevant memories, experiences, or knowledge to construct their answers. In the second approach, the participants decide whether to answer from a factual or an attitudinal perspective. The choice of approach is shaped by the participant’s background, previous experiences, and their perception of the pertinence and context of the items.

The first approach emerged as a central mechanism enabling participants to relate to the items and provide answers. Participants with extensive practical experience, such as nurses or dietitians, tended to feel more confident in their responses. Long-standing work in primary care, particularly involving tobacco cessation, provided these participants with a strong foundation for answering items about their self-efficacy in conducting conversations on lifestyle habits (QID6). Other participants described how tools such as MI (Motivational Interviewing), FYSS (Physical Activity on Prescription), and FAR (Physical Activity Referral) supported their ability to answer items related to physical activity and dietary habits. However, this approach was not solely based on professional experience. Some participants reflected on how their personal experiences of changing their own health behaviours enhanced their ability to relate to the items (QID7).

The second approach varied depending on the participants’ knowledge and prior experience. The factual strategy was predominantly employed by participants with professional backgrounds that allowed them to base their answers on concrete facts. For example, participants explained how their work with motivational strategies for various lifestyle habits strengthened their confidence in adopting a factual strategy (QID8). Conversely, participants with less professional experience often relied on an attitudinal strategy. Rather than drawing solely on factual or theoretical knowledge, they related to the items through personal and lived experiences of lifestyle habits. This strategy was particularly evident in situations where relationship-building and communication were crucial for the success of the conversation. Similarly, some participants drew on personal experiences of unhealthy habits to build confidence in their ability to guide others. Participants also identified challenges in answering the items, particularly among those with limited experience in addressing lifestyle habits overall or within specific domains. Participants reported uncertainty in assessing their theoretical knowledge *versus* practical abilities and whether their competencies were sufficient or accurate. These challenges affected their self-assessment, sometimes leading to either overestimation or underestimation of their capabilities and, consequently, their ability to provide reliable answers (QID9).

### Participants’ evaluations of item quality and relevance

This category relates to the participants’ evaluations of item quality and perceived relevance, response alternatives, missing items, layout issues, and overall suitability of the instrument.

#### Appraisal of clarity and relevance

Participants generally found the items to be both clear and relevant (QID10). Some described the instrument as quick and straightforward to complete, while others noted that certain items required more careful reflection. The similarity of items, together with their organisation of the instrument according to different lifestyle habits, was seen as helpful in facilitating use. Some participants felt that the items effectively encompassed the relevant lifestyle habits, while others suggested the inclusion of additional sections on stress and sedentary behaviour to better reflect contemporary societal issues.

The structure of certain items was described as slightly awkward but nonetheless functional. Participants stated that these minor issues were not disruptive, emphasising that the overall coherence of the instrument was more important than perfect phrasing. While some items appeared to be biased (framed either positively or negatively), it was suggested that they should instead be posed neutrally to ensure inclusivity and openness. To achieve a more holistic perspective, participants recommended the inclusion of items addressing both health benefits and health risks in each section, thereby integrating both salutogenic and pathogenic viewpoints (QID11). Items concerning ‘advice’ were highlighted as requiring clarification; for instance, using the term alone might suggest simple advice; however, care must be taken not to overcomplicate the phrasing around different levels of counselling to avoid misunderstandings.

Although the items were considered to adequately address discussions on lifestyle habits, participants suggested adding an item in each domain about referrals to other professionals to further enhance the instrument. Generally, the instrument was perceived as being framed from an expert-driven perspective. However, this could be nuanced by introducing an initial section prior to the lifestyle habit domains, focusing on conversational approaches. This could include items about person-centred care, the participant’s ability to create an environment where the patient feels comfortable speaking, and their ability to listen in a non-judgmental manner.

The open-ended item was appreciated but was also found to be ambiguous (QID12). Participants were unsure whether it applied to all sections or only to the last section on eating habits. Suggestions were made to either include an open-ended item in each section or clarify that the existing question was independent of the specific topics.

Participants also emphasised the importance of including more specific and contextualised items. For instance, items about a patient’s motivation and perceived health benefits should take into account socio-economic factors and previous attempts to change habits, as these elements profoundly influence an individual’s readiness for change. Similarly, items addressing the participant’s ability to recognise when and how to refer patients to other professionals, such as dietitians or addiction specialists, were notably absent but would add value. In the same vein, more detailed items on motivational strategies and how participants might employ these to support patients would further enhance the instrument’s relevance.

#### Appraisal of response alternatives

Regarding response alternatives, participants generally perceived the response alternatives as clear, relevant, and easy to distinguish. They engaged in evaluative reasoning to determine which alternative best aligned with their interpretation of the item content. Furthermore, the structure of the alternatives, such as fixed choices instead of a free scale and the use of an even number of categories, was appreciated, as it encouraged participants to take a definitive stance (QID13). Participants highlighted the importance of presenting items in a natural flow, beginning with habit mapping and followed by assessments of motivation, health benefits, and strategies for change. They suggested that a more intuitive structure would better reflect how real-life conversations typically unfold.

#### Appraisal of layout

In terms of layout, participants suggested that the presentation of response options could be improved by placing explanatory labels, such as ‘1 = Very uncertain’ and ‘4 = Very certain’, directly on each row rather than only in the instructions. This would reduce the need for participants to repeatedly refer back to the scale, though it might also make the instrument visually denser, potentially overwhelming some individuals (QID14). Overall, participants emphasised the value of a design that strikes a balance between clarity and simplicity while facilitating an efficient and intuitive response process.

Regarding content editing, beyond the response mechanics, participants respondents suggested that while the instrument is generally effective for identifying and discussing lifestyle habits, there is room for refinement to better reflect the realities of clinical practice. They felt that the logical flow of items could be further optimised (QID15). Items that presume a linear process of motivation or change were seen as unrealistic, given the complex and dynamic nature of behaviour change in real-world scenarios.

While the current instrument is regarded as thoughtfully designed, participants suggested additional refinements to improve its usability and pertinence. They proposed providing a clearer explanation of its purpose, emphasising that it is intended as a tool to identify needs and foster growth rather than to judge shortcomings. This clarification could help to reduce any perceived pressure or discomfort participants may feel during the process.

From a design perspective, the instrument’s layout and language were generally seen as well-constructed (QID16). However, participants identified certain inconsistencies, particularly in the transition between Swedish and English, which detracted from the overall cohesion. A more unified linguistic approach would strengthen the instrument’s professionalism. Headings, such as ‘Confidence in Knowledge’ and ‘Confidence in Ability’, could be positioned more prominently to improve navigation, while minor design inconsistencies, such as variations in the size of response box, should be addressed to eliminate distractions.

Although the use of colour to distinguish sections was appreciated, participants cautioned against excessive use, as this could make the design feel overwhelming. They suggested using subtle shading to guide the eye without detracting from the simplicity of the overall structure. Some participants also noted that densely packed text and instructions could hinder readability and proposed either spreading out the information or reformatting the instructions to improve the layout.

Participants appreciated the concise and clear instructions, which were considered particularly important given that the questionnaire targets a broad audience, including younger participants. Simplicity was emphasised as crucial to prevent participants from feeling overwhelmed or ignoring the instructions. Overly lengthy or detailed descriptions risk discouraging participants from reading the text, potentially leading them to complete the SELC quickly without adequate reflection on the items.

## Discussion

Qualitative pre-testing interviews were in this study used to support the validation of participants’ interpretations of and responses to SELC 28. Through Qualitative pre-testing interviews, the items can be adjusted according to the participants’ assessments. This process improves the instrument as a whole, making it easier to understand and more relevant for the end-users. When designing an instrument, there is always the risk that participants may not interpret the items as intended or may answer them differently from what was intended [27,28]. Therefore, three aspects of validity will be discussed: content validity, face validity, and user validity.

### Content validity

Content validity relates to the assessment of whether the instrument’s items adequately cover the intended constructs, which in this study was self-efficacy, e.g. a task specific confidence, in theoretical knowledge and practical ability to counsel patients, or clients, about lifestyle habits. Content validity also relates to whether the outcome measure is comprehensible to the intended target population [24,29]. To assess the intended constructs effectively, it is essential that no pivotal aspects of the phenomenon are omitted. Furthermore, it is vital that the included items really are relevant and accurately capture the intended phenomenon [30].

This is often assessed by independent experts, who evaluate the relevance of each item according to both the complete instrument and the relevance to the intended end-users [31]. The participants in the present study were selected for their expertise in the phenomena under investigation. Participants were included either as members of the target population, such as university nursing students, or as independent experts, including university teachers in health-related subjects and healthcare clinicians working with lifestyle counselling. By including both the target population and independent experts, rich data about the phenomenon was obtained, ranging from content and relevance to technical structure and phrasing of each item [32]. Interviews are often used to qualitatively assess content validity [24] and provide helpful knowledge about how participants interpret and engage with items [32,33]. As Wenemark [32] highlights, understanding how participants interpret and engage with instrument items is closely related to their motivation, since the perceived relevance and clarity of the items can influence both response quality and willingness to participate.

In the present study, participants stated that the items were generally relevant and comprehensible. However, some items caused confusion in their interpretation. For example, participants reported difficulties in distinguishing between knowledge and ability, which led to a revision of the instrument, so that it instead stated ‘theoretical knowledge’ and ‘practical ability’. By making these changes, there was better alignment between the intended meaning of the concepts and the participants’ interpretation. Similarly, participants highlighted the connotations associated with that the concept of ‘mapping’. While the intended meaning of the word was a one-to-one conversation, participants interpreted the word as implying something large-scale and distant. The concept was therefore revised to ‘identifying’, which more clearly emphasises the relational aspect. The interviews thus served to uncover misalignments between the participants’ interpretation of the items and their original intent, allowing for subsequent modifications [34]. The participants also emphasised the importance of situating the items within a context. When interpreting the items, they often considered a third person, and the understanding varied depending on who this third person was, i.e. the intended recipient of the counselling. The temporal and purpose-specific nature of content validity has similarly been highlighted in previous research [35].

### Face validity

The second aspect of validity, face validity, relates to the participants’ immediate assessment of whether they can determine what a specific item is intended to assess. This is an important aspect to ensure that participants perceive both the specific item and the instrument as a whole as relevant [31], which in turn motivates them to actually answer it [36]. When participants perceive the language of the item as difficult to understand and answer, they experience a higher perceived cognitive burden. Conversely, when items are clear and easy to understand and answer, the cognitive burden is low, which increases the likelihood of accurate responses [37]. Reducing cognitive burden is, therefore, an important aspect when developing an instrument.

One way of reducing cognitive burden and facilitating participants’ comprehension of the items is to avoid long or complex sentences, as well as unfamiliar concepts. In this study, participants noticed that the term knowledge could refer both to personal knowledge and to a broader, more general understanding. This ambiguity made them unsure of the intended meaning and increased cognitive burden. To improve comprehension, they suggested clarifying the phrase by adding ‘my’ in conjunction with knowledge, hence stating ‘my knowledge’. This clarification would make the question clear and decrease the cognitive burden. In addition, the term knowledge was related to another comprehension difficulty, as it was perceived to overlap with the term ability. When knowledge was interpreted as something more than just theoretical knowledge, such as tacit knowledge, it became difficult to distinguish from the concept of ability, thereby increasing cognitive burden. One way to try to reduce this confusion was, again, to clarify the terms as ‘theoretical knowledge’ and ‘practical ability’. However, due to this conceptual overlap, there was an increased risk of participants either underestimating or overestimating their knowledge/ability, which could potentially weaken validity.

### User validity

User validity relates to how accurately and effectively participants are able to interpret and apply the test output in practice, which, in turn, influences their motivation to respond [38]. Qualitative pre-testing interviews can complement psychometric testing by refining item relevance and helping ensure the items are practical to answer [24], given participants’ trade-off between the time required to retrieve item-related information and the perceived utility of the instrument [32,33]. The results show that participants’ previous experiences influenced their perceived time burden. Some participants had extensive professional experience in practising lifestyle counselling. This made it easier to access information, allowing them to rely on a factual response strategy and thus reducing the time required. Other participants drew on personal experiences of unhealthy habits, which affected their confidence in guiding others, and led them to rely on a more attitudinal response strategy. The results also show that the design and overall structure of the instrument, particularly the similarity between the different lifestyle habits, as highlighted by the participants, helped to reduce the time burden. Similarly, participants emphasised that having fixed response options instead of free scales, along with response options that were clear and easily distinguishable, facilitated the responding process.

The participants found the items to be relevant, which affects the assessment of utility *versus* time burden. However, they proposed suggestions for refining the instrument even further, in order to improve its usability and relevance. This included clarifying the purpose of the tool, emphasising its roles in identifying needs and fostering growth rather than assessing shortcomings. Such changes were not, however, included in SELC 20 + 20, as there is always a consideration to be made between reducing the questionnaire length and being comprehensive. Nevertheless, feelings of distress, such as lack of confidence in one’s knowledge or ability, could potentially have a negative impact on motivation to complete the instrument [32].

An important result was that participants perceived the instrument as expert-driven, which highlights a critical tension in individual-level lifestyle counselling. This aligns with previous research emphasizing that counseling is most effective when grounded in person-centered approaches and tailored to persons’ own motivations and life circumstances [3]. Rather than promoting directive interventions, the SELC instrument may therefore be best understood as a reflective tool to support professionals in assessing their readiness to engage in such person-centered conversations.

## Methodological strengths and limitations

This study qualitatively explored the SELC 28 instrument through qualitative pre-testing interviews with strategically selected participants. To strengthen the methodological rigor of the study, particular attention was paid to content validity during both study design and analysis. In line with a functional approach to content validity [23], the analysis focused on how participants understood and evaluated the clarity, relevance, and representativeness of the questions in relation to the intended construct.

Qualitative pre-testing, as recommended in scale development research [24], was used to identify potential weaknesses in wording, response alternatives, and overall item suitability. This approach strengthens evidence related to content validity by examining whether the items are perceived as adequate. The interviews were conducted in an interactive manner to allow in-depth exploration of participants’ interpretations and evaluations of the items. While this approach provided focused insights into item comprehension and appraisal, it may have limited the identification of unanticipated issues beyond those discussed during the interviews.

The number of participants (*n* = 8) falls within the recommended range for qualitative pre-testing interviews; however, it still constitutes a limitation. Although the sample was purposefully selected to capture variation in experience and perspective, recruitment was confined to a specific geographical area in southern Sweden and limited to certain educational and clinical contexts. As a result, content validity may have been affected, as certain relevant interpretations or contextual nuances might not have been captured. To mitigate this limitation and enhance the generalisability of the results, external validity was addressed through the deliberate inclusion of three distinct participant groups: students, university teachers, and clinicians. The diversity of their perspectives encompassed a broad spectrum of experiences and interpretative frameworks, thereby expanding the contextual relevance of the results [39]. Moreover, testing the instrument with participants possessing both theoretical knowledge and practical expertise in health care ensured that its content was both relevant and comprehensible to the intended audience. This is particularly critical given that the instrument is intended for use in both educational and clinical settings [40].

To enhance intercoder reliability and ensure the objectivity of the analysis, coding was conducted independently by multiple researchers, followed by comparative discussions of their interpretations. This process reduced the risk of subjective bias [41]. In addition, the use of an interview guide containing probing questions supported consistency and replicability in data collection. Although the results are context-bound, the study design allows replication in similar contexts, thereby supporting both the reliability and objectivity of the instrument’s validation. To further ensure reliability, the analytic process was thoroughly documented, coding was cross-checked, and researchers engaged in consensus discussions to refine interpretations, following the recommendations of O’Connor and Joffe [41]. An initial cross-tabulation served as a form of pre-analysis, offering an overview of how participants’ reflections related to each item and to the four analytical categories. This contributed to analytic consistency and transparency. The use of a structured categorization matrix further supported a focused and systematic approach to the analysis, thereby strengthening reliability in line with established contentanalysis methodologies [42].

## Conclusion

The purpose of this study was to quality test the SELC 28, and the qualitative pre-testing interviews proved to be a valuable way of gaining insights into and identifying difficulties in the participants’ interpretation of the instrument and its fit to what it aimed to measure. Together with previously published psychometric testing of the SELC 28 [22], these results contribute to the evaluation of the instrument’s validity and applicability. SELC 28 can serve as a tool for managers and educators to design interventions aimed not only at enhancing self-efficacy in domains that need further improvement but also at strengthening and maintaining areas in which confidence is already established. The participants provided suggestions for improvements regarding design and layout. For example, they proposed formulating items in a neutral rather than a positive or negative manner, which may be helpful when designing instruments. The same goes for other suggestions related to layout, such as emphasising a salutogenic purpose of the instrument early on to avoid misunderstandings, which could otherwise lead to feelings of shortcomings or distress. The participants emphasised the need to include the intended recipient and their specific circumstances when considering the items’ applicability. This situational sensitivity could be useful not only for participants but also for the development of new instruments. The instrument offers a way to assess healthcare professionals’ self-efficacy in lifestyle counselling, supporting both their motivation and their decision to engage in this activity. Similarly, the instrument can be used in educational initiatives to help healthcare professionals feel more confident and prepare them for their future work. Consequently, the instrument and its results may serve as a starting point for further discussions and mutual reflection, as a way to decide on concrete local interventions and practices. A recommendation for further research is to develop a model or supporting material to be used in the subsequent dialogue on self-efficacy and possible actions.

## Supplementary Material

Additional file.docx

## Data Availability

The raw data can be obtained from the corresponding author upon reasonable request.
