# Supplementary material for: Exploring item comprehension and evaluation in the SELC scale: a qualitative pre-testing study
Source: Scand J Prim Health Care. 2026 Apr 27;44(1):2663295. doi: 10.1080/02813432.2026.2663295 (PMC13123070; doi:10.1080/02813432.2026.2663295)
Supplement: Additional file.docx [file IPRI_A_2663295_SM7525.docx]

**Self-Efficacy in Lifestyle Counselling scale – SELC-28**

The lifestyle habits of tobacco use, alcohol consumption, physical activity and eating habits are of great importance for both health promotion, as well as the prevention and treatment of non-communicable diseases. This questionnaire assesses self-efficacy regarding your knowledge and ability to counsel individuals about their lifestyle.

| For each question, choose how confident you are in your KNOWLEDGE and ABILITY to counsel individuals within the respective lifestyle habits. | Circle one number based on the  following answer options:  1. I am very insecure in my…  2. I am insecure in my…  3. I am sure of my…  4. I am very sure of my… | | | | | | | | |
| --- | --- | --- | --- | --- | --- | --- | --- | --- | --- |
|  | …confidence in **knowledge**: | | | | … confidence in **ability**: | | | | |
| Tobacco |  | | | |  | | | | |
| 1. Mapping of tobacco use | 1 | 2 | 3 | 4 |  | 1 | 2 | 3 | 4 |
| 1. Health effects of tobacco use | 1 | 2 | 3 | 4 |  | 1 | 2 | 3 | 4 |
| 1. Advice about tobacco | 1 | 2 | 3 | 4 |  | 1 | 2 | 3 | 4 |
| 1. Assessment of motivation for tobacco cessation | 1 | 2 | 3 | 4 |  | 1 | 2 | 3 | 4 |
| 1. Motivational strategies for tobacco cessation | 1 | 2 | 3 | 4 |  | 1 | 2 | 3 | 4 |
| Alcohol | | | | | | | | | |
| 1. Mapping of risky alcohol use | 1 | 2 | 3 | 4 |  | 1 | 2 | 3 | 4 |
| 1. Health effects of reducing alcohol consumption | 1 | 2 | 3 | 4 |  | 1 | 2 | 3 | 4 |
| 1. Advice about alcohol | 1 | 2 | 3 | 4 |  | 1 | 2 | 3 | 4 |
| 1. Assessment of motivation to reduce alcohol consumption | 1 | 2 | 3 | 4 |  | 1 | 2 | 3 | 4 |
| 1. Motivational strategies for reducing alcohol consumption | 1 | 2 | 3 | 4 |  | 1 | 2 | 3 | 4 |
| Physical activity | | | | | | | | | |
| 1. Mapping of insufficient physical activity (everyday exercise, physical training, muscle strengthening, balance training and sedentary lifestyle) | 1 | 2 | 3 | 4 |  | 1 | 2 | 3 | 4 |
| 1. Health effects of physical activity | 1 | 2 | 3 | 4 |  | 1 | 2 | 3 | 4 |
| 1. Advice about physical activity | 1 | 2 | 3 | 4 |  | 1 | 2 | 3 | 4 |
| 1. Assessment of motivation for increased physical activity | 1 | 2 | 3 | 4 |  | 1 | 2 | 3 | 4 |
| 1. Motivational strategies for increasing physical activity | 1 | 2 | 3 | 4 |  | 1 | 2 | 3 | 4 |
| Eating habits | | | | | | | | | |
| 1. Mapping of unhealthy eating habits | 1 | 2 | 3 | 4 |  | 1 | 2 | 3 | 4 |
| 1. Health effects of healthy eating habits | 1 | 2 | 3 | 4 |  | 1 | 2 | 3 | 4 |
| 1. Advice about eating habits | 1 | 2 | 3 | 4 |  | 1 | 2 | 3 | 4 |
| 1. Assessment of motivation to improve eating habits | 1 | 2 | 3 | 4 |  | 1 | 2 | 3 | 4 |
| 1. Motivational strategies for healthy eating habits | 1 | 2 | 3 | 4 |  | 1 | 2 | 3 | 4 |
| 1. Additional comments for the above questions:   ___________________________________________________________________________________________________________________________________________________________________________________________________________________________________________________________________________________________________________________________ | | | | | | | | | |

***Thank you for your participation!***

© 2024 Sara Alenius, Marie Rask, Petra Nilsson Lindström, Marie Nilsson, Lina Behm. The instrument may only be used in its entirety and with reference to the authors.
